# Emperors in Hiding: When Ice-Breakers and Satellites Complement Each Other in Antarctic Exploration

**DOI:** 10.1371/journal.pone.0100404

**Published:** 2014-06-25

**Authors:** André Ancel, Robin Cristofari, Peter T. Fretwell, Phil N. Trathan, Barbara Wienecke, Matthieu Boureau, Jennifer Morinay, Stéphane Blanc, Yvon Le Maho, Céline Le Bohec

**Affiliations:** 1 Université de Strasbourg, Institut Pluridisciplinaire Hubert Curien, Strasbourg, France; 2 Centre National de la Recherche Scientifique, UMR 7178, Strasbourg, France; 3 Centre Scientifique de Monaco, LEA-647 BioSensib, Monaco, Principality of Monaco; 4 British Antarctic Survey, Cambridge, United Kingdom; 5 Australian Antarctic Division, Kingston, Tasmania, Australia; Institute of Ecology, Germany

## Abstract

Evaluating the demographic trends of marine top predators is critical to understanding the processes involved in the ongoing rapid changes in Antarctic ecosystems. However, the remoteness and logistical complexity of operating in Antarctica, especially during winter, make such an assessment difficult. Satellite imaging is increasingly recognised as a valuable method for remote animal population monitoring, yet its accuracy and reliability are still to be fully evaluated. We report here the first ground visit of an emperor penguin colony first discovered by satellite, but also the discovery of a second one not indicated by satellite survey at that time. Several successive remote surveys in this coastal region of East Antarctica, both before and after sudden local changes, had indeed only identified one colony. These two colonies (with a total of *ca.* 7,400 breeding pairs) are located near the Mertz Glacier in an area that underwent tremendous habitat change after the glacier tongue broke off in February 2010. Our findings therefore suggest that a satellite survey, although offering a major advance since it allows a global imaging of emperor penguin colonies, may miss certain colony locations when challenged by certain features of polar ecosystems, such as snow cover, evolving ice topology, and rapidly changing habitat. Moreover our survey shows that this large seabird has considerable potential for rapid adaptation to sudden habitat loss, as the colony detected in 2009 may have moved and settled on new breeding grounds. Overall, the ability of emperor penguin colonies to relocate following habitat modification underlines the continued need for a mix of remote sensing and field surveys (aerial photography and ground counts), especially in the less-frequented parts of Antarctica, to gain reliable knowledge about the population demography and dynamics of this flagship species of the Antarctic ecosystem.

## Introduction

The emperor penguin (*Aptenodytes forsteri*), a flightless seabird endemic to Antarctica, was probably first seen on James Cook’s second voyage, in 1773–1774 Yet the first breeding colony was only discovered in 1902 during the first of Scott’s Discovery Expeditions [1]. For the colonies that could be reached from research stations, population trends usually involved ground counts [2], [3]. The census of those not accessible by ground surveys was usually obtained using aerial photography [4]. The first estimation from space was carried out in 2005 and 2006 for the western Ross Sea area [5], while the first global survey was performed in 2009 [6], [7]. Today, 52 colonies of emperor penguins have been identified (Fretwell and Trathan, unpublished data), all of them being distributed along the coastline of Antarctica between 64°S and 77°S [8]–[11]. However, our present knowledge of the biology of the emperor penguin falls far behind the picturesque notoriety of this iconic species. The breeding behaviour and phenology of the species have been described from only a few sites, e.g. from the Dion Islands [12] and Pointe Géologie archipelago [13], usually settled in the vicinity of overwintering research stations. Indeed, most colonies are just too remote from permanently occupied research stations, and because of the unique breeding cycle of this species (in which egg laying, hatching, and early chick rearing all occur during the austral winter; [13]), ground visits to most breeding colonies are virtually impossible for most of the breeding season [2]. Yet, because the French station in Adélie Land, Dumont d’Urville, has been located next to the Pointe Géologie colony, this is the only colony which has been visited almost every day for more than 45 years [13]–[22]. However, it has been shown that demographic studies extrapolated from the colony-level can be strongly misleading as they tend to give too much weight to local stochastic events [23].

The recently developed satellite-based remote-sensing methods [5]–[7] are a major breakthrough. By detecting faecal deposits on the sea ice in satellite images, it is possible to locate emperor penguin colonies. However, present-day satellite technology is constrained in Polar Regions as the darkness of the polar winter and the frequent cloud cover during winter and early spring limit window of opportunity for satellite-surveys. Further, wind-blown snow may obscure the faecal signal. Therefore, to expand our knowledge of this species beyond the boundaries of the few available local observations, it is critical to corroborate and calibrate these remote-sensing methods through ground visits with detailed and precise observations of breeding colonies.

The ground survey of the Mertz Glacier area was initiated within this context and became logistically possible in November 2012. The existence of an emperor penguin colony near the Mertz Glacier had been suspected for almost a hundred years, and Mertz himself perished in an attempt to locate the colony. More recently, during August 1999, sightings of thousands of emperor penguins traversing the northern part of the Mertz Glacier tongue [24] confirmed that a colony must be located close to the glacier.

By using Landsat and QuickBird2 Very High Resolution Landsat imagery, confirmation of the colony site at 66°54′S, 146°37′E at the tip of the Mertz glacier tongue was obtained in November 2009 [6], [7]. The high resolution of the satellite imagery allowed counting 4,781 pairs at the time of image acquisition. However, before a field survey could take place, the Mertz Glacier tongue calved in mid-February 2010 presumably inducing the colony to relocate or merge with another colony. New satellite images obtained since then suggested that the birds might attempt to breed on different sites depending on the year. Still, since the exact colony location and colony size appeared to change between successive observations, ground-based assessments were difficult and needed to be conducted over several seasons.

Our objective was to validate the presence of one or more emperor penguin colonies near the Mertz Glacier, to locate them, to estimate their population size (breeding adults) and breeding success (number of chicks), and to obtain other biological information.

## Materials and Methods

Animal ethics were not an issue as we counted the birds from aerial pictures. No manipulation or experimentation was conducted on live birds and care was taken to avoid any disturbance. The French Polar Institute (IPEV) and the Terres Australes et Antarctiques Françaises (TAAF) are the authorities that issued the permit to visit the colonies.

During the 2012–2013 austral summer, the French Polar Institute’s resupply ship *MSS L’Astrolabe* visited the Antarctic coastline near to the Mertz Glacier. *L’Astrolabe’s* classification is ice class 1A super, the highest class of vessels that can operate in difficult polar conditions without the assistance of an ice breaker. On November 1^st^ and 2^nd^, using a Eurocopter Squirrel B-3 single-engine helicopter, aerial surveys were conducted along the northern edge of the Mertz Glacier in an attempt to locate the new breeding sites of the emperor penguin colonies. Aerial photographs, used for recording penguin location and for counting the chicks and adults, were taken obliquely in clear weather from an altitude of 300 meters, with a 35-mm Canon 40D digital camera fitted with a 200 mm f/4 lens.

Aerial photographs were stitched together to form photo-mosaics in PTGui© software, and both chicks and adults were counted separately using the count tool in Photoshop CS5©. This was repeated by two different observers. The total number of individuals was estimated using two different methods to provide a probable range for each colony census: 1) under the hypothesis that inter-annual climatic effects are largely dominant over fine-scale local effects, we made the assumption that breeding success is approximately similar in nearby colonies for any given year. We therefore estimated the number of breeding pairs from the early November chick-counts using the population parameters determined from the nearby Pointe Géologie colony (66°40′S, 140°01′E) over the whole breeding season; 2) following Budd [2], who proposed that, based on a census at several different colonies, adult counts between October and November equate to approximately one-third (26% to 40%) of the total adult population, independently of the actual breeding success. For comparison, the Pointe Géologie colony was counted at different times, from photographs, using the same methodology. The total number of incubating males in mid-June was taken as a proxy for the total number of breeding pairs: and chicks were counted in early November, to allow comparison with the census of the Mertz colonies.

To avoid potential disturbance, no low altitude flights were made over or near to the Mertz colonies, and a brief landing was made *ca.* 1 km from the breeding sites to minimize further disturbance. Each colony was visited by 4 or 5 people on foot, and surveyed for approximately 3 hours each. No direct ground counts of chicks or adults were made during the visits due to the lack of any satisfactory vantage point. Observations focused on assessing the habitat, chick abundance and health (though no direct measures were taken in order to avoid unnecessary disturbance). Observations were also made to search for flipper-banded individuals, potentially originating from the Pointe Géologie colony, located at only *ca.* 250 km to the west.

To find colony locations, satellite observations were made using freely available reduced resolution “quicklooks” from the commercial satellite provider DigitalGlobe (https://browse.digitalglobe.com/imagefinder/). These images have an approximate on-the-ground resolution of ∼10 m per pixel. Colonies were located visually from the images by detecting the brown signature of the bird’s faecal stains on the ice. Once located, raw Very High Resolution imagery with a resolution of 0.5 m per pixel was acquired. This resolution allows areas of penguins to be differentiated from guano using a multivariate supervised classification method [5], [7].

## Results

### Habitat and Chick Census

#### First or eastern colony

**Figure 1:**
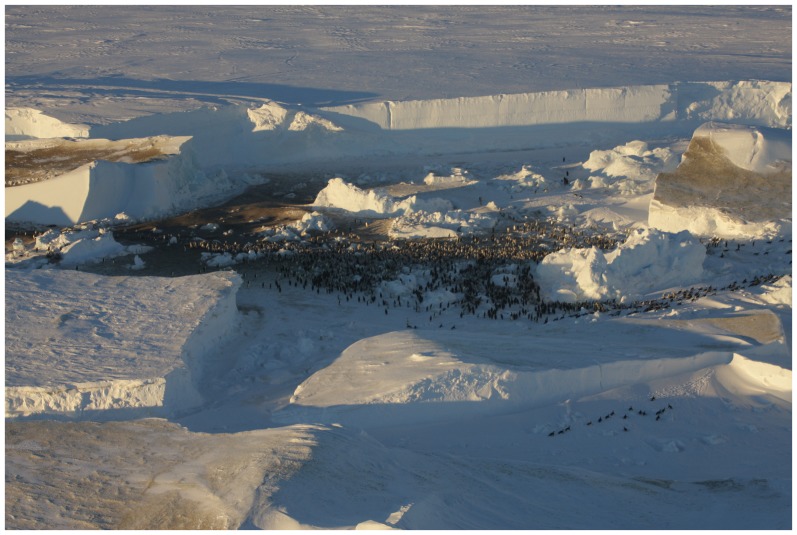
Eastern emperor penguin colony. Aerial view of the eastern emperor penguin colony (67°19′S, 145°52′E) showing adults and chicks present on November 1^st^, 2012. Photograph by Robin Cristofari, from an altitude of *ca.* 1,000 feet.

**Figure 2:**
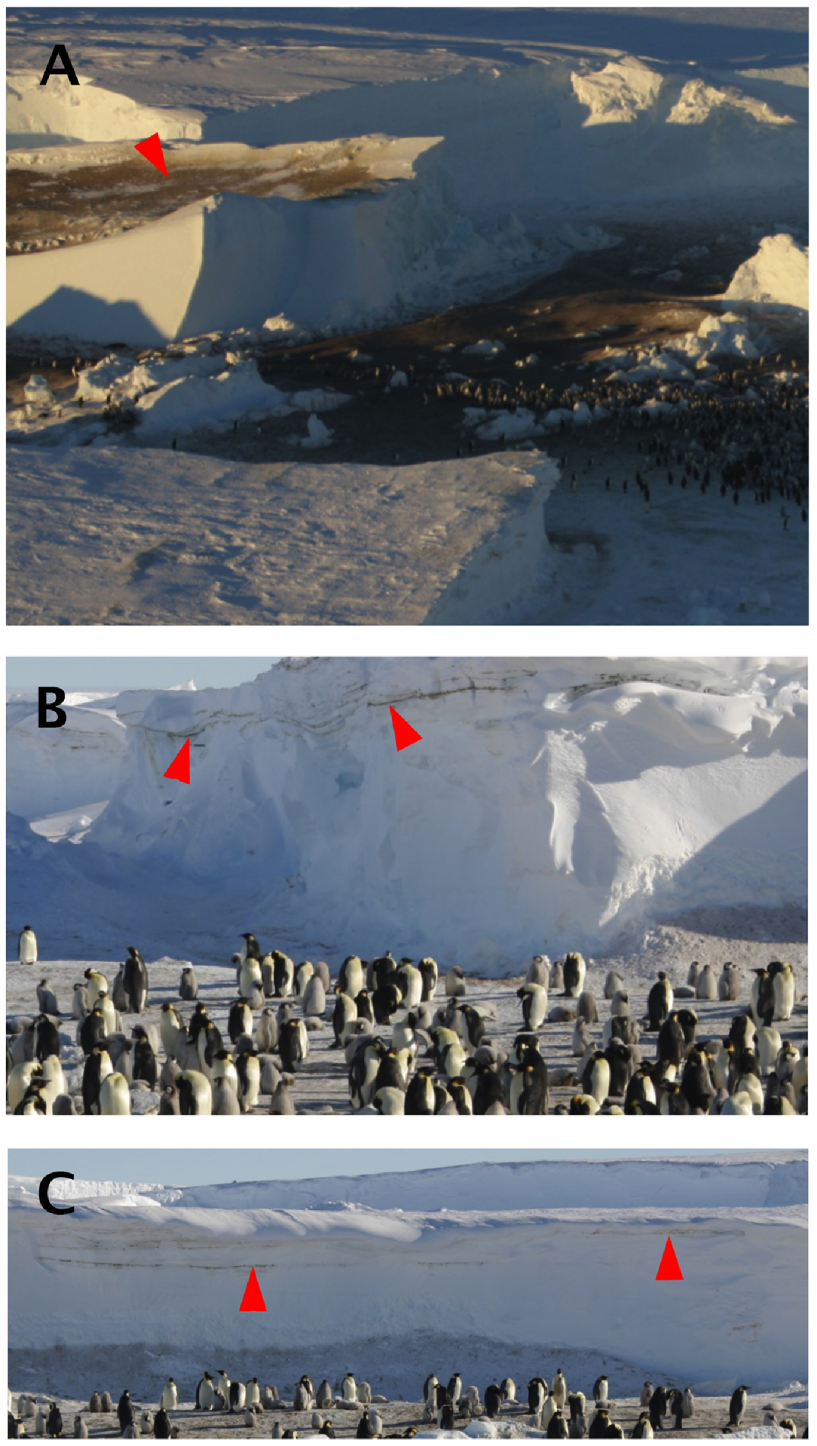
Eastern emperor penguin colony. **A.** Location of the eastern colony (67°19′S, 145°52′E) on the sea-ice at the time of our visit and previous locations of the colony on top of icebergs, *ca.* 10 m high, covered with guano (arrow). **B & C.** Layers of droppings (arrows) covered by several layers of snow indicate abundant snow-covered episodes during the breeding season. Photographs by Robin Cristofari.

The existence of a breeding colony of emperor penguins near the Mertz Glacier was confirmed and located at 67°19′S, 145°52′E on November 1^st^, 2012 (Figure 1). The eastern colony was breeding on fast ice amidst grounded icebergs, 250 km east of Dumont d’Urville research station. The colony was composed of numerous sub-groups, which appeared to have moved short distances, as confirmed by the clustered guano deposits. The topography of the breeding site was very uneven with numerous icebergs embedded in the sea ice, offering protection from the prevailing high winds. Layers of droppings were found between layers of snow on top of the icebergs (Figure 2), providing evidence of numerous snow-fall episodes. Many icebergs, *ca.* 10-meter high, were also covered with guano, thus demonstrating that breeding birds had spent some time on the top of these icebergs (Figure 2). This colony had three former incubation areas, identified by stained sea ice, numerous egg shells and carcasses of newly hatched chicks. During our 3-hour visit, no birds other than penguins, and no sign of predation (e.g. predated or scavenged carcasses) were found. Two Weddell seals *Leptonychotes weddelli* were sighted along tidal cracks in the vicinity. Our survey yielded a count of 1,750 downy chicks, and 1,520 adults. Approximately 300 dead chicks, ranging in age from a few days (frozen) to 3-month-old (both frozen and fresh) were found. Most of them were found at the incubation sites, and on the top of small icebergs.

#### Second or western colony

**Figure 3:**
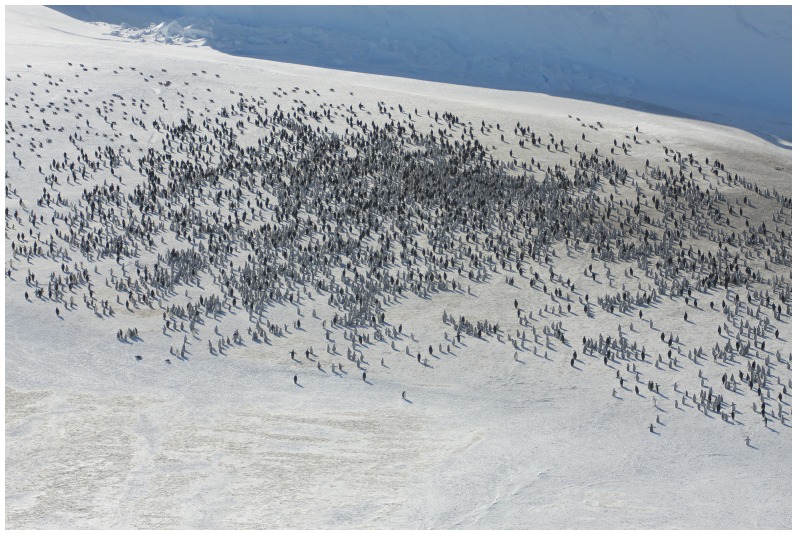
Western emperor penguin colony. Aerial view of the western emperor penguin colony (67°14′S, 145°30′E) showing adults and chicks present on November 2^nd^, 2012. Photograph by Robin Cristofari, from an altitude of *ca.* 1,000 feet.

On November 2^nd^, 2012, a second colony was discovered by chance from an altitude of *ca*. 500 m during a helicopter flight involved in resupply operations. This western colony was located on a large and flat fast ice sheet extending from the Mertz glacier, *ca.* 20 km west of the first colony, at 67°14′S, 145°30′E (Figure 3). This colony was bordered on the north-west by a high ice cliff of the Mertz glacier. Numerous large tabular icebergs were grounded in the fast ice, south of the colony. This habitat was similar to that generally found in the Ross Sea area, characterized by stable fast ice, nearby open water and access to fresh snow [25]. The site was poorly sheltered from the wind. The colony comprised one single group of *ca.* 3,980 downy chicks, and 2,300 adults. From empirical observation, these chicks seemed bigger, and better fed, than those of the eastern colony. Three Antarctic skuas *Catharacta maccormicki* and one giant petrel *Macronectes giganteus* were sighted flying over the colony. Only 17 dead chicks and one abandoned egg were found during our 3-hour survey, some of them partly buried. The ice surface was of heavily compacted and abraded snow, and it is therefore most likely that more dead chicks were buried beneath the snow surface. Guano traces were restricted to the area currently occupied by the birds, and its direct surroundings. Due to these difficult conditions, we were unable to precisely locate the incubation area. As for the first colony, we did not observe any individuals bearing flipper-bands.

### Extrapolation to Total Colony Size

Our average 2012 breeding season count for the Pointe Géologie colony was *ca.* 3,200 breeding pairs, and we counted *ca.* 2,500 chicks in early November 2012, which gives a pair-to-chick ratio of *ca.* 1.28. Applying this ratio to the chick counts for the Mertz colonies, we estimated the population size to be *ca.* 2,300 breeding pairs for the eastern colony, and *ca.* 5,100 for the western colony (Table 1).

**Table 1:** 2012 census of the Mertz emperor penguin colonies.

	Latitude S	Longitude E	Chicks	Adults	Chick-to-adult ratio	Pairs (from chicks)^a^	Pairs (from adults)
**Eastern colony**	67°19′	145°52′	1,750	1,520	1.15	∼2,300	1,900–2,900
**Western colony**	67°14′	145°30′	3,980	2,300	1.73	∼5,100	2,900–4,400

a Breeding pairs were obtained by applying a pair-to-chick ratio of *ca.* 1.28 obtained from the emperor penguin colony at Pointe Géologie, Adélie Land.

Moreover, a total of 1,864 individual adults were counted at the Pointe Géologie colony on October 31^st^, 2012, which implies a total count of *ca*. 2,330 to 3,580 breeding pairs according to Budd’s method. This is consistent with our average 2012 winter count of *ca*. 3,200 breeding pairs, thus allowing us to apply this method to the Mertz Glacier census.

Adult counts from photographs were 1,520 for the eastern colony and 2,300 for the western colony, respectively (Table 1). When applying Budd’s method [2], the estimated population size was 2,300 breeding pairs for the first colony (ranging from 1,900 to 2,900) and 3,500 for the second (from 2,900 to 4,400), hence a total of *ca.* 5,800 breeding pairs for the Mertz emperor penguin population.

## Discussion

### Strength and Shortcomings of the Remote-sensing Technique

Our study confirmed sightings made 15 years ago of thousands of emperor penguins going back and forth at the northern part of the tongue of the Mertz Glacier, suggesting the presence of a colony in this area [24]. Moreover, our ground visit to the two Mertz colonies demonstrates the reliability of the remote-sensing method developed by researchers [5], [6]. Although the method successfully identified several previously known emperor penguin colonies, this is the first time that the existence of three colonies identified solely from satellite images have been accurately corroborated in the field: two at the new edge of the Mertz Glacier in late 2012 (present study) and one on Princess Ragnhild coast (69°54′S, 27°09′E) in early 2013 (visit http://www.antarcticstation.org/for more information).

However, the locations from satellite surveys of the two colonies at the edge of the Mertz Glacier were not determined during the same year, therefore suggesting the existence of only one colony in this area. This fact suggests that further refinement of the present-day remote-sensing method may be advantageous. Only the western colony was located in December 2011, while the eastern one remained invisible. In contrast, only the eastern colony was located in 2012. Failure to identify both colonies at the same time may be attributed to different factors. To locate colonies, the remote-sensing method relies on guano staining on the sea-ice surface, and heavy snow cover may obliterate the signal. The western colony, which could not to be detected just a few weeks before our visit, was largely covered by snow during the last weeks of winter. Hence, almost no guano stains, abandoned eggs, or dead chicks were found. The location of this colony along a high glacier edge may also have kept it hidden from the satellite, if the image was taken at too shallow an angle. In addition, a previous study [26] has shown that the density of emperor penguin colony can vary considerably, i.e. from 2 to 9 birds per square meter, and this within only a few hours. This means that census estimates based on colony area may be inaccurate in some circumstances due to variability in density and to inter-annual variability. This suggests that this colony was not the only one to be missed by the 2009 and 2012 satellite surveys, and that more colonies remain to be discovered in other parts of the continent. Although not always possible each season due to cloud cover and the availability of the satellite, our results suggest that satellite surveys should be conducted repeatedly and combined with field surveys to ensure that colonies are not missed. Our paper shows that to allow confidence in satellite observations, a multi-temporal/multi-year approach has to be used to ensure that breeding sites are not missed due to heavy snowfall, deep shadows or topographic features such as ice cliffs. If possible, these observations should be backed up with aerial or ground counts as the limited special resolution of satellite imagery results in large inherent variances when calculating breeding populations. Innovative cameras combined with biologging would also be very useful to complete the satellite survey. For instance, since the satellite survey based on 2009 imagery, four more emperor penguin colonies have been discovered: two on the West Ice Shelf detected by aerial survey [27], and two others on the West Ice Shelf and near the Jason Peninsula identified by satellite survey [28].

### Influence of the 2010 Calving on the Mertz Emperor Penguin Colonies

**Figure 4:**
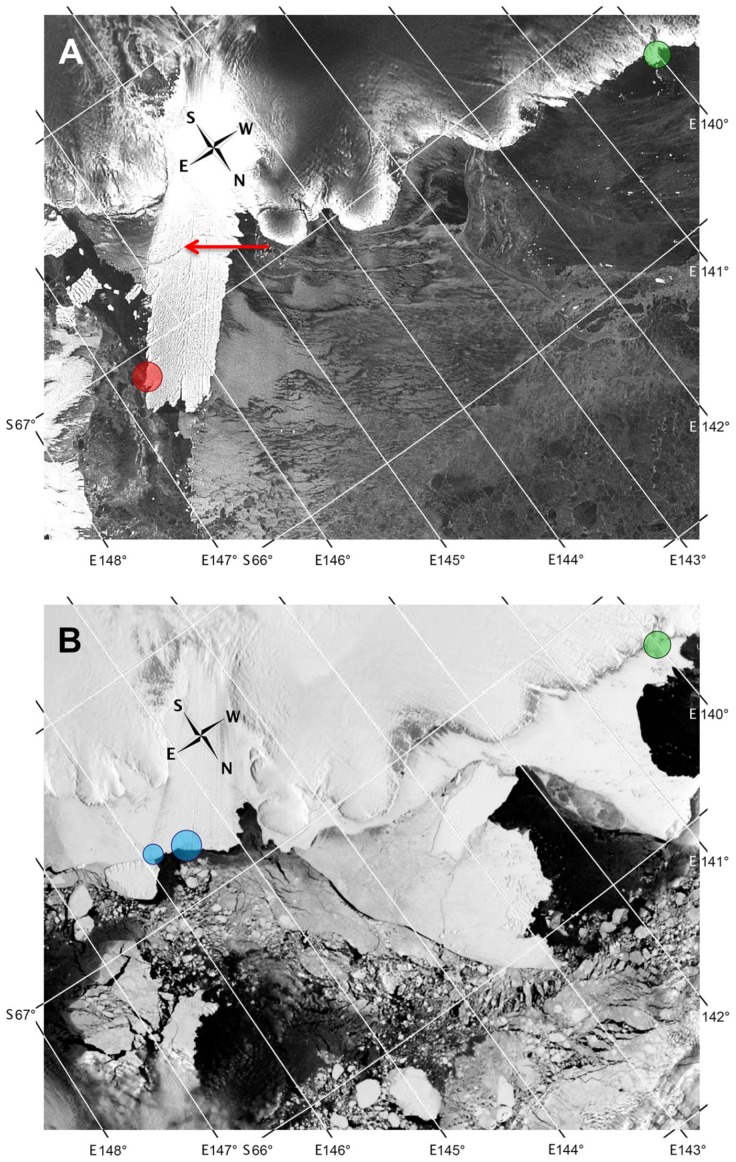
Satellite images of the Mertz Glacier. Circle area is proportional to colony size. Continent is on top, open sea at the bottom. **A.** Estimated location of the Mertz emperor penguin colony on November 13^th^, 2009 (red circle) on the eastern flank of the Glacier tongue. A large crack in the Mertz Glacier is visible (red arrow). The green circle corresponds to Pointe Géologie colony. **B.** Location of the two new colonies of emperor penguins on December 3^rd^, 2012 (blue circles), 2 years after the 2010 calving of the Glacier tongue. The berg (overall length of 80 km and a width of 40 km) broke off the Mertz Glacier after being rammed by another iceberg. The green circle corresponds to Pointe Géologie colony. Images downloaded from the USGS website (https://lta.cr.usgs.gov).

Due to the discrepancy between the coordinates given by satellites (66°54′S, 146°37′E; [7]) and our land based ones (67°19′S, 145°52′E and 67°14′S, 145°30′E), we may assume that the colony initially detected from space in 2009 has been split into two colonies. Following the calving of the Mertz Glacier, the birds may indeed have attempted to settle in new favourable surroundings. The two new colonies are separated by 20 km along the new northern edge of the Glacier (Figure 4). Taking into account the proximity of the two breeding sites, these colonies might however reunite in the future, especially if one or other of the sites eventually proves to be more reliable for breeding and/or foraging.

All satellite surveys conducted up to now, before and after the Mertz calving, had concluded the presence of a single emperor penguin colony, even if grouped into three close sub-colonies in 2009, in the Mertz Glacier area. It may also be that two nearby colonies already existed at the current location(s) before the 2010 calving. This is supported by the fact that the best population estimate of the Mertz Glacier colony located by Fretwell et al. [7] was estimated to be 4,781 adults (or 5,976 pairs if we considered that 80% of the total breeding population was present during the satellite survey [7]), a figure which is relatively close to our assessment of 5,100 breeding pairs for the western colony alone (Table 1) but lower than the sum of the two colonies we found (7,400 breeding pairs; Table 1). In such a scenario, the second colony we discovered might be the one found before at the nearby Ninnis Glacier [29], which would have been missed in all further surveys (see [6]).

### Influence of the Breeding Location at Fine-spatial Scale

It is important to note that the two colonies appeared to differ slightly in their breeding stage. The chick-to-adult ratio was higher in the western colony than in the eastern one (1.73 *vs.* 1.15, respectively: Table 1). This result may be interpreted as a difference in breeding success, with higher chick mortality in the eastern colony than in the western one. This hypothesis is supported by the much higher number of dead chicks in the eastern colony. Moreover, while the two colonies were visited on two consecutive days, the western colony appeared more advanced in its breeding cycle, as indicated by the generally better health and bigger size of the chicks than in the eastern colony. Finally, the western colony was much closer to the open water than the eastern one at the time of our visit, and a recent study [30] has shown that the distance between an emperor penguin colony and the open water may tend to correlate negatively with breeding success.

Budd [31] proposed that “each rookery can be regarded as a compromise, and not always a very successful one, between the emperor’s sometimes conflicting requirements of safety from sea-ice breakouts, shelter, and access to food”. Finding a suitable trade-off may well be an arduous matter of trial-and-error, since inter-annual variations make it difficult for the birds to evaluate rapidly the consistency of ice conditions at a particular location. Therefore, after the loss of their original habitat, some birds are probably breeding in a sub-optimal location, which may explain the difference in breeding success between neighbouring and possibly related colonies.

### Status of the Emperor Penguins Census

Emperor penguins breed almost around the entire coastline of the Antarctic Continent [7], [9], [32]–[34]. Their known breeding distribution extends from Snow Hill Island (64°31′S, 57°26′W) to Gould Bay (77°43′S, 47°41′E) in the Antarctic Peninsula, and Cape Crozier on Ross Sea (77°28′S, 169°19′E). The Snow Hill Island [9], [35] and Gould Bay [9], [31] colonies are the most northerly and the most southerly known emperor penguin colonies, respectively. Colony sizes vary from more than 20,000 pairs (Coulman Island, Cape Washington, Halley Bay) to just a few hundred pairs (Umbeashi Rock, Amundsen Bay, Fold Island, Cape Crozier). The two largest known colonies are in the Ross Sea: Cape Washington with *ca.* 24,000 pairs and Coulman Island with *ca.* 28,000 pairs [25].

The closest colonies to the Mertz Glacier are at Pointe Géologie (66°40′S, 140°01′E) and Davis Bay (69°21’S, 158°29′E). While *ca.* 2,500 chicks of emperor penguins were raised at the Pointe Géologie colony during the 2012 breeding season, the two new colonies together numbered *ca.* 5,700 chicks. Since a pair of emperor penguins may only successfully raise one chick per year, the population of breeding emperor penguins in this area of Antarctica can therefore be estimated, based on these chick counts, to more than *ca.* 16,400 breeding adults, about a fourth more than previously estimated [7]. This count represents a minimum estimate for the breeding population, given uncertainty about the mortality rate of chicks prior to our visit.

Because of the persistence of the sea ice, few ships are able to reach the Antarctic coasts before post-breeding adults disperse from their colonies between December and early January. Except for colonies close enough to research stations, during the breeding season (from March-April to December-January, depending on the latitude), access to colonies is difficult or impossible due to adverse weather and/or extensive pack ice [25]. Consequently, many emperor penguin colonies have never been counted and some have presumably not even yet been discovered. Furthermore, in addition to the Mertz colonies, another colony, on the Princess Ragnhild coast and for which location was reported in 2009 by satellite images [7], was first visited in early January 2013 by Belgian scientists, who surveyed *ca.* 20,000 adults, compared to 6,870 adults detected from satellite survey [7]. The current global emperor penguin population can therefore be estimated to be, at least, *ca.* 260,000 pairs from 52 breeding colonies [7 and present study], or *ca*. 25% more than estimated only 15 years ago [35].

Over the past five years satellite surveys have proved a very effective method for finding new emperor penguin colonies. However, as our visit to the western colony demonstrated, the nitrogen-signature of snow-covered droppings around colonies that underwent numerous snowfalls, and the shade of an ice front indicate that at certain times of year, under challenging environmental conditions it is virtually impossible to detect some colonies from satellite images. Importantly, several colonies have not been observed since they were first reported. This problem, of missing colony locations by satellite survey may be solved by taking multiple satellite images within a breeding season and especially towards the end of the breeding season when the guano is more apparent. Multi-temporal and multi-year satellite surveys, backed up with confirmation and further ground truthing from aerial or ground based counts are essential if we wish to fully understand the population demography and dynamics of emperor penguins. As a consequence, we can still hope to discover more emperor penguin colonies in the future if further investigations are conducted along the Antarctic coast.
